# Characterization of the complete mitochondrial genome of *Corynespora cassiicola* (Pleosporales: Dothideomycetes), with its phylogenetic analysis

**DOI:** 10.1080/23802359.2019.1662753

**Published:** 2019-09-06

**Authors:** Cheng Chen, Qiang Li, Rongtao Fu, Jian Wang, Zhonghan Fan, Xuejuan Chen, Daihua Lu

**Affiliations:** a Institute of Plant Protection, Sichuan Academy of Agricultural Sciences, Chengdu, Sichuan, P. R. China;; b Key Laboratory of Integrated Pest Management on Crops in Southwest, Ministry of Agriculture, Chengdu, Sichuan, P. R. China;; c College of Pharmacy and Biological Engineering, Chengdu University, Chengdu, Sichuan, P.R. China

**Keywords:** *Corynespora cassiicola*, mitogenome, phylogenetic analysis

## Abstract

*Corynespora cassiicola* is a well-known plant pathogen with a broad host range and diverse lifestyles. In this study, we presented the complete mitochondrial genome (mitogenome) of *C. cassiicola* for the first time. It has a total length of 40,752 bp, which encodes 17 protein-coding genes (PCGs), 2 ribosomal RNA genes (rRNA), and 27 transfer RNA (tRNA) genes. The nucleotide composition of the mitogenome is: A (36.24%), T (34.62%), G (15.74%), and C (13.39%). Phylogenetic analysis revealed that *C. cassiicola* has a close relationship with *Didymella pinodes* from *Didymellaceae*.

*Corynespora cassiicola* is a type species of the genus *Corynespora* (Dixon et al. 2009), which is an Ascomycetes fungus with a broad host range and diverse lifestyles and exists in more than 400 plants (Farr and Rossman 2019). This fungus is mostly known as a necrotrophic plant pathogen, mainly distributed in the tropics and subtropics (David et al. 2018). The pathogen infects many agricultural crop plants, especially rubber, tomato, cucumber, cowpea, papaya, and soybean, causing huge economic losses (Dixon et al. 2009). The pathogen has also been reported to cause diseases in humans (Huang et al. 2010; Yamada et al. 2013). At present, *C. cassiicola* has been proved to have many kinds of pathogenic types and there are no clear geographical and host boundaries (Dixon et al. 2009; Marine et al. 2014). The mitochondrial genome of *C. cassiicola* reported here will allow further investigations on the taxonomy, phylogenetics, and evolution of this important pathogen.

The specimen was isolated from a brown leaf spot disease of cultivated kiwifruit (*Actinidia chinensis*) in Sichuan Province, China (103.24 E; 30.15 N). The sample was identified as *C. cassiicola* according to Cui et al. (2015). The strain was stored in Sichuan Academy of Agricultural Sciences (No. SAAS_cca1). Genomic DNA of obtained mycelia was extracted (DNA Kit D3390-00, Omega Bio-Tek, Norcross, GA) following the manufacturer’s instructions and was stored in the sequencing company (BGI Tech, Shenzhen, China). We constructed the sequencing libraries with purified DNA using the NEBNext Ultra II DNA Library Prep Kit (NEB, Beijing, China). Whole genomic sequencing was performed using an Illumina HiSeq 2500 Platform (Illumina, San Diego, CA, USA). We performed quality control and de novo assembly of the mitogenome according to Li (2019). The SPAdes 3.9.0 (Bankevich et al. 2012) was used to de novo assemble the *C. cassiicola* mitogenome with the obtained clean reads; gaps between contigs were filled using MITObim V1.9 (Hahn et al. 2013). The MFannot tool was used for mitogenome annotation of *C. cassiicola,* combined with manual corrections. tRNAs were predicted with the tRNAscan-SE 2.0 (Lowe and Chan 2016).

The complete mitogenome of *C. cassiicola* (GenBank accession No. MN239093) was assembled as a closed-circular molecule of 40,752 bp in length. The circular mitogenome contains 17 protein-coding genes(PCGs), 27 tRNA genes, and 2 ribosomal RNA genes (rnl and rns). The overall nucleotide composition of *C. cassiicola* mitogenome is as follows: A (36.24%), T (34.62%), G (15.74%), and C (13.39%).

To validate the phylogenetic position of *C. cassiicola,* we constructed a phylogenetic tree based on 15 typical PCGs from *C. cassiicola* and other 18 species from Dothideomycetes, Eurotiomycetes, Leotiomycetes, and Sordariomycetes, respectively. The Bayesian inference (BI) method was used for phylogenetic analysis based on the combined gene datasets with the MrBayes 3.2.6 program (Ronquist et al. 2012). As shown in the phylogenetic tree (Figure 1), the taxonomic status of the *C. cassiicola* based on combined mitochondrial gene dataset exhibits a close relationship with *Didymella pinodes* from *Didymellaceae*.

**Figure 1.**
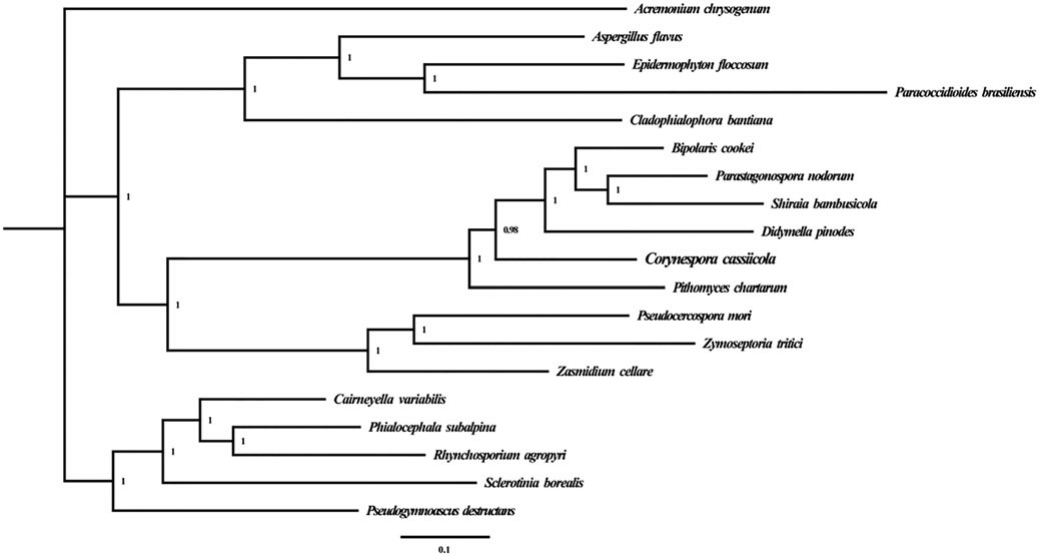
Phylogenetic relationship of 19 species based on Bayesian inference analysis of the combined mitochondrial gene set (15 core protein-coding genes). Node support values are Bayesian posterior probabilities (BPP). Mitogenome accession numbers used in this phylogeny analysis: *Didymella pinodes* (NC_029396), *Zymoseptoria tritici* (NC_010222), *Zasmidium cellare* (NC_030334), *Pithomyces chartarum* (NC_035636), *Shiraia bambusicola* (NC_026869), *Parastagonospora nodorum* (NC_009746), *Pseudocercospora mori* (NC_037198), *Cairneyella variabilis* (NC_029759), *Phialocephala subalpina* (NC_015789), *Pseudogymnoascus destructans* (NC_033907), *Rhynchosporium agropyri* (NC_023125), *Sclerotinia borealis* (NC_025200), *Bipolaris cookei* (MF784482), *Acremonium chrysogenum* (NC_023268), *Cladophialophora bantiana* (NC_030600), *Aspergillus flavus* (NC_026920), *Epidermophyton floccosum* (NC_007394), *Paracoccidioides brasiliensis* (NC_007935), *Corynespora cassiicola* (MN239093).
